# Novel effects of Ras-MAPK pathogenic variants on the developing human brain and their link to gene expression and inhibition abilities

**DOI:** 10.21203/rs.3.rs-2580911/v1

**Published:** 2023-02-21

**Authors:** Bhavana Rai, Paige Naylor, Monica Siqueiros Sanchez, Max Wintermark, Mira Raman, Booil Jo, Allan Reiss, Tamar Green

**Affiliations:** Stanford University School of Medicine; Stanford University School of Medicine; Stanford University School of Medicine; Stanford University; Stanford University; Stanford University; Stanford University; Stanford University

## Abstract

The RASopathies are genetic syndromes associated with pathogenic variants causing dysregulation of the Ras/mitogen-activated protein kinase (Ras-MAPK) pathway, essential for brain development, and increased risk for neurodevelopmental disorders. Yet, the effects of most pathogenic variants on the human brain are unknown. We examined: 1. How Ras-MAPK activating variants of *PTPN11*/*SOS1* protein-coding genes affect brain anatomy. 2. The relationship between *PTPN11* gene expression levels and brain anatomy, and 3. The relevance of subcortical anatomy to attention and memory skills affected in the RASopathies. We collected structural brain MRI and cognitive-behavioral data from 40 pre-pubertal children with Noonan syndrome (NS), caused by *PTPN11* (*n* = 30) or *SOS1* (*n* = 10) variants (age 8.53 ± 2.15, 25 females), and compared them to 40 age- and sex-matched typically developing controls (9.24 ± 1.62, 27 females). We identified widespread effects of NS on cortical and subcortical volumes and on determinants of cortical gray matter volume, surface area (SA) and cortical thickness (CT). In NS, we observed smaller volumes of bilateral striatum, precentral gyri, and primary visual area (*d’s*<−0.8), and extensive effects on SA (*d’s*>|0.8|) and CT (*d’s*>|0.5|) relative to controls. Further, SA effects were associated with increasing *PTPN11* gene expression, most prominently in the temporal lobe. Lastly, *PTPN11* variants disrupted normative relationships between the striatum and inhibition functioning. We provide evidence for effects of Ras-MAPK pathogenic variants on striatal and cortical anatomy as well as links between *PTPN11* gene expression and cortical SA increases, and striatal volume and inhibition skills. These findings provide essential translational information on the Ras-MAPK pathway’s effect on human brain development and function.

## Introduction

The Ras/mitogen-activated protein-kinase (Ras-MAPK) pathway plays a crucial role in regulating neural cellular processes such as growth, proliferation, and differentiation affecting the development of the central nervous system^1,2^. In humans, pathogenic variants causing dysregulation of the Ras-MAPK pathway are associated with a group of genetic syndromes called RASopathies. Noonan syndrome (NS) is the most common RASopathy, occurring in 1:1000–2500 live births and displaying autosomal dominant inheritance^3^. NS is characterized by a broad spectrum of cognitive deficits and phenotypic features such as symptoms associated with attention deficit hyperactivity disorder (ADHD), autism spectrum disorder, oppositional defiant disorder, and anxiety disorders^4,5^. The study of NS provides a unique opportunity to examine Ras-MAPK regulatory effects on neurodevelopment.

Gain-of-function pathogenic variants in specific genes encoding components of the Ras-MAPK pathway, including *PTPN11* and *SOS1*, are associated with NS. Pathogenic variants of the *PTPN11* gene are associated with approximately 50% of NS cases^3^. The *PTPN11* gene encodes Src homology-2 domain-containing protein tyrosine phosphatase-2 (SHP2) protein, a major regulatory protein tyrosine phosphatase in the Ras-MAPK pathway. *PTPN11* variants constitutively activate SHP2, leading to downstream upregulation of the Ras-MAPK cascade^3,6^. In mouse models, SHP2 activation increased neurogenesis and decreased astrogenesis^7^, while decreasing the number of myelinated axons and causing abnormal myelination in white matter^8^. In a preliminary structural neuroimaging study including children with *PTPN11* (*n* = 12) and controls (*n* = 12), ages 4 to 11, we reported reductions in bilateral striatal volume, surface area (SA) of temporal regions, and cortical thickness (CT) in limbic regions as well as CT increases in frontal regions^9^.

High variability in cognitive deficits observed in children with NS may be due to the different pathogenic variants associated with the syndrome^10^. However, no study to date has examined brain development in children with *SOS1* variants, which are associated with approximately 10% of NS cases^11^. The *SOS1* gene encodes a guanine nucleotide exchange factor that activates Ras and downstream Ras-MAPK signaling; *SOS1* gain-of-function variants further enhance this activation^3^. *SOS1* has been shown to play a role in neurite outgrowth by stimulating nerve growth factor^12^, which is expressed at high levels in neonatal cortical tissue, and activates the Ras-MAPK pathway through interacting with N-methyl-D-aspartate (NMDA) glutamate receptors in the neonatal cortex^13^. Previous studies comparing physical phenotypes of individuals with *PTPN11* or *SOS1* have found that pulmonary stenosis, atrial septal defects, short stature, and significant developmental delays are less prevalent in *SOS1* relative to *PTPN11*. These differing phenotypes suggest that disruptions caused by *PTPN11* and *SOS1* variants at the molecular level may have overlapping, yet distinct, features^14,15^. While preliminary assessments indicate distinct effects on cognition^10^, differences in brain phenotypes between *PTPN11* and *SOS1* have not previously been investigated. Thus, evaluating *PTPN11* and *SOS1* groups separately in our analyses allows us to take the first step in elucidating these differences.

In this study, we had three aims: first, to confirm our preliminary findings^9^ of NS effects on subcortical regions in a larger sample size and expand our investigation to include the developing human cortex. Second, we tested whether NS variants (*PTPN11* and *SOS1*) have different effects on brain anatomy. Third, using gene expression levels from postmortem adult human brains (Allen Institute for Brain Science; http://www.brain-map.org), we explored whether *PTPN11* expression levels correlate with NS effects on regional brain anatomy^16,17^. Finding correlations between NS gene expression levels and aberrant brain development would unravel which brain regions are more susceptible to gain-of-function variants in the Ras-MAPK pathway. Finally, we examined correlations between aberrant brain development in NS and performance in attention and executive function to support the prediction that brain-based findings have behavioral consequences.

## Methods And Materials

### Participants

Participants included 40 children with NS associated with either *PTPN11* (19 female) or *SOS1* (6 female) variants, ages 4.43–12.3 years (mean 8.53 ± 2.15), and 40 age- and sex-matched TD controls, ages 4.05–11.9 years (mean 9.24 ± 1.62). 12 *PTPN11* and 10 TD participants from our previous study cohort were included^9^. In this cohort, there are an additional 28 NS participants (18 *PTPN11*, 10 *SOS1*) and 30 TD controls not included in our previous study. Further details regarding participant recruitment and exclusion criteria are included in Supplementary Material. Medication history and Tanner staging were assessed by an experienced physician (TG) and are summarized in Table 1 and Supplementary Material. Parents or legal guardians provided informed written consent for their child’s participation in the study; participants over age 7 submitted an additional written assent. Study protocols were approved by the Stanford University School of Medicine Institutional Review Board and followed during all study components.

### MRI

Participants received behavioral training in a mock MRI scanner familiarizing them with the MRI environment in an effort to minimize motion-related artifacts. All participants were scanned using a GE Healthcare Discovery 3.0 T whole-body MR system (GE Medical Systems, Milwaukee, WI) with a standard 8-channel head coil at Stanford University Lucas Center for Imaging. Additional details about the pulse sequence and image quality check are presented in Supplementary Material.

### Structural Analysis (Freesurfer)

Cortical reconstruction and volumetric segmentation of cortical and subcortical structures were performed with FreeSurfer image analysis suite, version 5.3 (http://surfer.nmr.mgh.harvard.edu/). Bias field correction methods in the SPM8 software toolkit (http://www.fil.ion.ucl.ac.uk/spm) were utilized for preprocessing of structural MRI scans prior to processing through the FreeSurfer pipeline. Brain surfaces for each hemisphere were parcellated into 34 distinct regions defined by gyral and sulcal boundaries^18,19^ and for each region, gray matter volume (GMV), surface area (SA) of the gray-white matter boundary, and mean cortical thickness (CT) are calculated; these values are presented in Table 3. Trained raters with inter-rater reliability of ≥ 0.95 (intraclass correlation coefficient) visually inspected cortical reconstruction and segmentation output from FreeSurfer as a second quality control check of scan usability and performed manual corrections as needed per FreeSurfer Tutorial guidelines (http://surfer.nmr.mgh.harvard.edu/fswiki/FsTutorial).

### Cognitive And Behavioral Assessment

General intelligence measures were collected through administering Wechsler Preschool and Primary Scale of Intelligence (WPPSI-III) or Wechsler Intelligence Scale for Children (WISC-IV) (Table 1)^20,21^. Attention, executive function, and memory were assessed with A Developmental NEuroPSYchological Assessment (NEPSY-II). Behavior Assessment System for Children (BASC-2) was administered to assess hyperactivity and inattention symptoms as well as to provide a comprehensive description of psychopathology.

### Allen Human Brain Atlas

Gene expression data were obtained from the Allen Human Brain Atlas (AHBA) (consisting of 3,702 postmortem brain samples from 6 donors), which provides an anatomically comprehensive examination of gene expression in the healthy adult human brain (Allen Institute for Brain Science; http://www.brain-map.org)^16^. Complete microarray gene expression datasets for both hemispheres of all 6 donors (5 males and 1 female; age range 24–57 years) were extracted for all cortical regions; right hemisphere data was only available for 2 out of 6 donors^16^. All donors were free of psychiatric drugs based on blood samples collected postmortem. First, for each brain tissue sample, mean average expression values of all *PTPN11* microarray probes were calculated. Samples were then mapped semi-automatically to 34 cortical regions in each hemisphere defined by the Desikan-Killiany atlas^19^, using the methods developed by French and Paus^17^. For each of the 68 cortical regions, *PTPN11* expression values were mean averaged across samples mapped to that specific region; median values were calculated for each region and each individual. Finally, *PTPN11* expression values were median averaged across the 6 donors to generate a single *PTPN11* expression value for each region. We then correlated the vector of *PTPN11* expression values across the 68 brain regions with between-groups effect sizes of FreeSurfer-computed morphometric measures (GMV, SA, and CT) for each of the 34 Desikan-Killiany regions per hemisphere. The currently available atlas for the developing brain is the BrainSpan atlas (Allen Institute for Brain Science; https://www.brainspan.org/), which includes gene expression data from brain tissue of typically developing donors across stages of human development (embryonic stages to age 40). However, only three of the donors are within the age range of our study (ages 4–12) and, unlike the Allen Human Brain Atlas, the BrainSpan atlas does not cover the entire cerebral cortex and provides gene expression values for only 11 out of 34 cortical regions segmented by FreeSurfer as designated by the Desikan-Killiany atlas^16,17,19^. To correct for spatial autocorrelation of structural imaging and transcriptomic data, we employed the null-spatial model^22^. We performed randomized cortical parcellations (1000 randomizations) by “spinning” the reconstructed sphere of the real cortical parcellation, thus preserving the spatial covariance of the data. By mapping transcriptomic samples to these randomized brain regions, we rebuilt gene expression matrices, which were used to generate null distributions. Similarly, we employed null models–null-random-gene and null-brain-gene–to test if the observed gene-brain correlations are specific to the *PTPN11* gene. Gene expression of same-sized gene sets were randomly selected (10,000 permutations) from approximately 20,000 genes included in Allen Human Brain Atlas for the null-random-gene model or from a subset of these genes consisting of genes overexpressed in the brain (relative to non-brain tissues in the body) for the null-brain-gene model. Next, we correlated expression levels from these gene sets to SA effect size in the corresponding brain regions to generate null distributions which we then used to re-evaluate the significance of the gene-brain correlations.

### Statistical Analysis

We performed all statistical analyses using The R Project for Statistical Computing (R) (http://www.r-project.org). We used unpaired *t*-tests to compare demographic characteristics and questionnaire scores between either the *PTPN11* or *SOS1* groups and the TD group (Table 1). To compare gray matter volume (GMV), SA, and CT between the *PTPN11* and TD groups, we conducted an analysis of covariance (ANCOVA) for each region-of-interest (ROI). We controlled for either total cortical tissue volume (referred to as total brain volume, TBV, in this study), total surface area (TSA), or weighted mean cortical thickness (WMT) for GMV, SA, and CT, respectively, in our regional analyses after finding significantly smaller TBVs in NS relative to controls to ensure that significant differences in regional morphometric measures cannot be attributed to smaller brain volume. For each ROI, we used GMV, SA, or CT measures as the dependent variable and diagnosis as the between-group factor, and age, sex, and either TBV, total SA, or mean CT, respectively, as covariates. Given the smaller sample of individuals with *SOS1* (*n* = 10), we used unpaired *t*-tests to compare each ROI’s GMV, SA, and CT to the TD group (*n* = 40). To control for TBV, total SA, and mean CT, for each ROI, we calculated residuals, derived after regressing out either TBV, total SA, or mean CT, respectively. We used these adjusted measures and performed unpaired *t*-tests between the two groups (*SOS1* and TD) for each ROI. In both analyses, the results (p-values) were adjusted for multiple comparisons using false discovery rate (FDR) correction^23^. For each ROI, we calculated effect sizes, with Cohen’s *d*, for *PTPN11* vs. TD and *SOS1* vs. TD after using the residual approach to adjust for TBV. To test convergence of NS subgroups on brain anatomy, we correlated (Pearson’s *r*) effect sizes vectors associated with each NS subgroup, generating 4 *r* values in total: *r*-subcortical, *r*-cortical GMV, *r*-SA and *r*-CT. Finally, observed *r* values were compared with null distributions of *r* values generated by repeating the analyses 2,000 times with permutation of subgroup genetic status. We explored brain-behavior correlations in the *PTPN11* and TD groups with and without controlling for TBV using Pearson correlations between striatal GMVs and NEPSY-II subtest scores in the following neurocognitive domains: Attention (Auditory Attention and Response Set) and Executive function (Inhibition – Naming, Inhibition, Switching); Memory and learning (Memory for Faces Delayed and Narrative Memory). We tested group differences between the NS and TD brain-behavioral correlations with Fisher tests. To increase our power to detect between-group differences and reduce number of comparisons, we calculated left and right striatal volumes by summing together caudate, putamen, and pallidum volumes on each side. In addition, we focused on attention, executive function, and memory domains which have previously been implicated in NS^24,25^ and only used measures within these domains that were compatible across our cohort’s age range (Table 1). For between-group analyses, we used FDR to adjust for multiple comparisons^23^.

## Results

We did not find differences in age or sex between the *PTPN11* and TD groups. We found a difference in age (*t*(13.7)=−2.58, *p* = 0.022), but not sex between the *SOS1* and TD groups (Table 1). For the *PTPN11* group, TBV (*t*(69)=−4.11, *p* = 0.00011, *d*=−0.98), total SA (*t*(69)=−2.38, *p* = 0.020, *d*=−0.55) and mean CT (*t*(69)=−4.19, *p* = 0.00010, *d*=−1.04) were smaller than those of the TD group. For the *SOS1* group, TBV (*t*(49)=−1.46, *p* = 0.17, *d*=−0.49), total SA (*t*(49)=−0.80, *p* = 0.44, *d*=−0.44), and mean CT (*t*(49)=−1.91, *p* = 0.078, *d*=−0.70) were not significantly smaller compared to the TD group. However, we observed relatively large effect sizes (all *d’s*<−0.44) in the *SOS1* group, indicating an overall effect of *SOS1* in the same direction as *PTPN11*. All images survived Euler number cut off − 217 assessing the FreeSurfer-compatible quality of images before manual editing^26^. However, we detected significant differences (*t*(78) = 2.244, *p* = 0.028) in cumulative Euler number between the NS (mean − 178.25±−73.57) and TD (mean − 145.25±−56.90) groups. To address group differences in cumulative Euler number, we conducted gold-standard manual edits on all images in FreeSurfer (see Methods and Materials).

### Noonan Syndrome Is Associated With Smaller Subcortical Volumes

**Figure 1:**
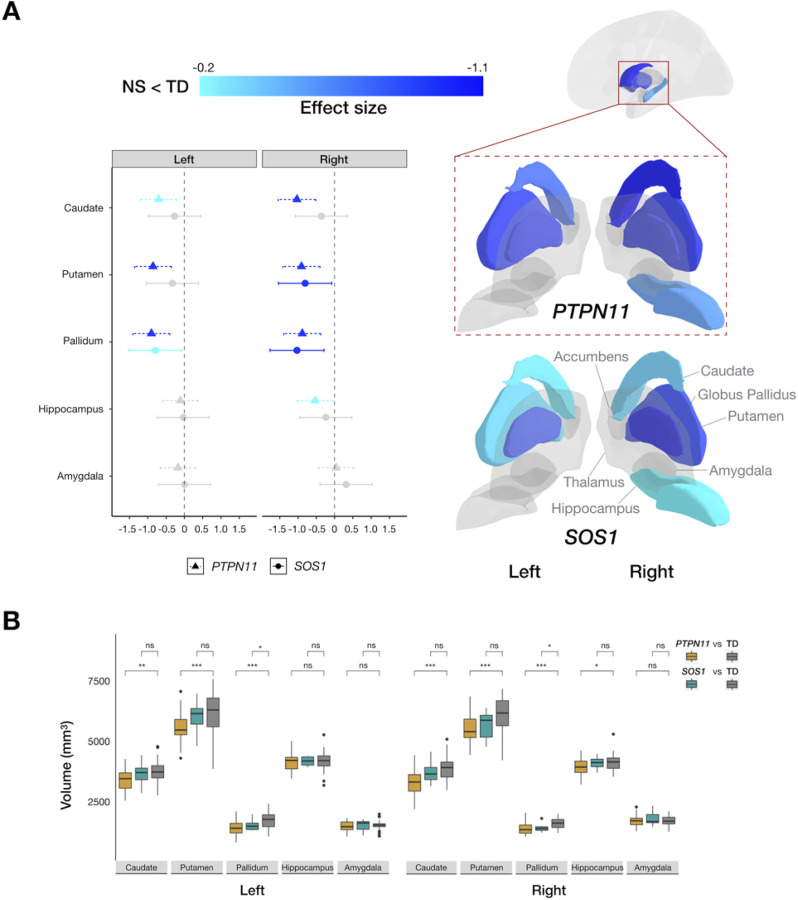
Subcortical structures affected by *PTPN11* and *SOS1* variants in Noonan syndrome. (A) Left: 95% confidence interval plot of *PTPN11*GMV effect sizes with cooler colors indicating larger negative values (NS < TD) and shapes and line types representing groups (*PTPN11* and *SOS1*); Right: Effects of *PTPN11* and *SOS1* variants in Noonan syndrome on subcortical anatomy indicated by effect sizes mapped onto a three-dimensional representation of bilateral subcortical brain regions. (B) Boxplots representing subcortical volumes in the *PTPN11*, *SOS1*, and TD groups. In *PTPN11*, we found smaller bilateral caudate (ANCOVA: left: *F*(1, 65)=10.11, *p*=0.0060, *d*=−0.70; right: *F*(1, 65)=22.61, *p*=0.00012, *d*=−1.03), putamen (left: *F*(1, 65)=17.28, *p*=0.00031, *d*=−0.86; right: *F*(1, 65)=21.01, *p*=0.00012, *d*=−0.91), and pallidum (left: *F*(1, 65)=20.79, *p*=0.00012, *d*=−0.91; right: *F*(1, 65)=19.60, *p*=0.00015, *d*=−0.88) and smaller right hippocampal GMV (F(1, 65)=6.071, *p*=0.029, *d*=−0.53) relative to the TD group. Similar to *PTPN11*, we found smaller pallidum GMV, bilaterally, (*t*-test: left: *t*(39)=−2.94, *p*=0.038, *d*=−0.80; right: *t*(39)=−3.74, *p*=0.012, *d*=−1.03) in the *SOS1* group compared to the TD group. Between-group differences are denoted for significance (*p<0.05, **p<0.01, ***p<0.001, ns: not statistically significant).

To evaluate the effect of NS on brain anatomy, we first examined its effect on GMV. For *PTPN11*, we found GMV reductions in bilateral striatal structures, specifically in the caudate, putamen, and pallidum. We also detected smaller right hippocampal GMV relative to the TD group (Fig. 1). Similarly, in the *SOS1* group, we found bilateral reduction in pallidum GMV compared to the TD group (Fig. 1). Using effect sizes to evaluate the clinical effect of *PTPN11* and *SOS1* variants on subcortical structures, we detected reduced subcortical volumes in both NS groups in a step-wise decrease pattern, with smaller effect (All *d’s*<−0.25) on striatal structures of *SOS1* and larger effect (All *d’s*<−0.7) of *PTPN11* compared to controls (Fig. 1).

#### Noonan syndrome affects gray matter volumes of precentral gyri and medial aspect of the occipital lobe

**Figure 2:**
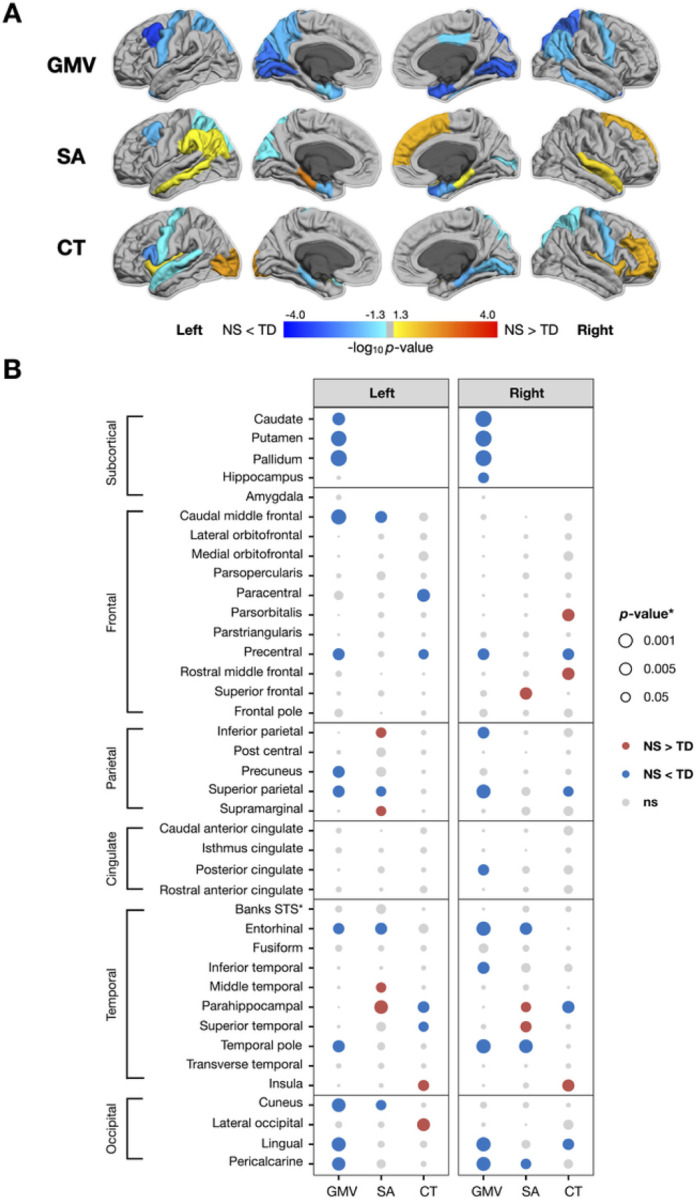
Significance (*p*-value) of changes in gray matter volume (GMV), surface area (SA), and cortical thickness (CT) in the *PTPN11* group. Dorsal aspects of the frontal and parietal lobes, as well as medial temporal and occipital regions, were particularly affected by *PTPN11* variants. (A) *p*-values of changes in gray matter volume (GMV), surface area (SA), and cortical thickness (CT) mapped to cortical ROIs with cooler colors indicating NS < TD and warmer colors indicating NS > TD, converted to −log_10_ (*p*) for visualization purposes. (B) *p*-values of changes in GMV, SA, and CT organized by hemisphere with rows for ROIs, columns for measures, dot color representing the direction of differences (blue: negative or NS < TD; red: positive or NS > TD), and dot size representing magnitude of *p*-values.

**Figure 3:**
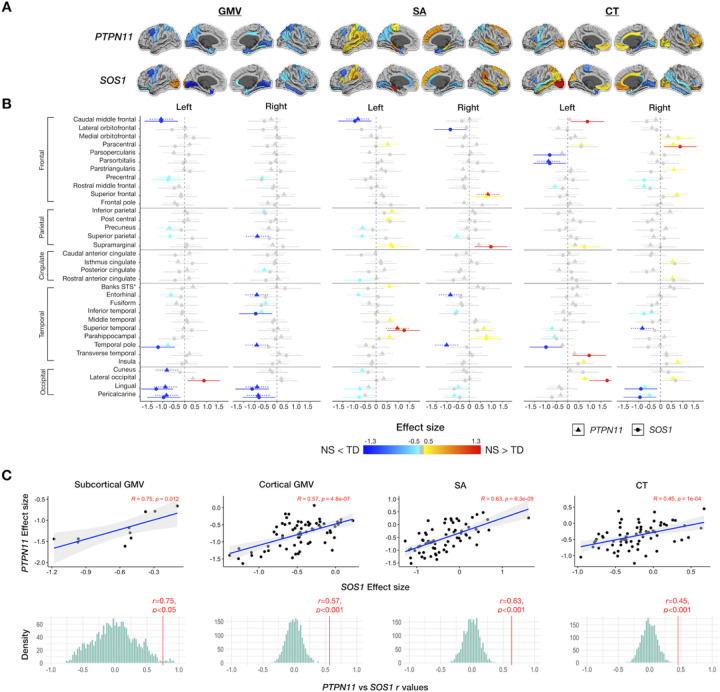
Effect sizes of gray matter volumes (GMV), surface area (SA), and cortical thickness in NS variants (*PTPN11* and *SOS1*). *PTPN11* and *SOS1* variants in Noonan syndrome affect cortical anatomy of similar cortical regions, generally in the same direction, with *PTPN11*having a more pronounced effect than *SOS1*. (A) Effect sizes of gray matter volume (GMV), surface area (SA), and cortical thickness (CT) mapped to cortical ROIs with cooler colors indicating NS < TD and warmer colors indicating NS > TD (B) 95% confidence interval plots of *PTPN11* and *SOS1* effect sizes organized by hemisphere and measure (GMV, SA, CT) with rows for ROIs, colors indicating direction of effect size (cooler colors: negative or NS < TD; warmer colors: positive or NS > TD, gray: non-significant values), and shapes and line types representing groups (*PTPN11* and *SOS1*) (C) Scatterplots illustrating the close coherence between effect sizes of NS status (*PTPN11* or *SOS1*) on subcortical and cortical gray matter volumes and gray matter determinants (SA and CT) between the *PTPN11* and *SOS1* groups and permutation testing distributions (with 2,000 null *r* values) demonstrating that observed correlations are significantly greater than null expectations.

We detected smaller regional GMV in *PTPN11* compared to the TD group, most prominently in the bilateral precentral gyri and medial aspect of the occipital lobe (All FDR *p*-values < 0.05) (Table S2; Fig. 2). Given that the *SOS1* group was smaller (*n* = 10) than the *PTPN11* group (*n* = 30), we lacked power to detect differences between these groups across the cortex. Therefore, to estimate the clinical effect of NS variants on the brain and compare these effects between NS groups, we used effect sizes. In general, we detected GMV decreases in a similar regional distribution for *PTPN11* and *SOS1* compared to controls (Fig. 3). For both NS groups, we observed large negative (All *d’s*<−0.8) effect sizes for GMV in the left caudal middle frontal gyri and medial aspect of the occipital lobe, compared to the TD group (Fig. 3).

#### Noonan syndrome is associated with surface area expansions in limbic regions and decreases in the frontal lobe

Next, we aimed to test whether the two determinants of cortical volume, SA and CT, are affected by NS. We observed SA decreases in bilateral entorhinal and left superior parietal cortices and SA expansion in the right frontal and bilateral temporal lobes in *PTPN11* compared to the TD group (All FDR *p*-values < 0.05) (Table S2; Fig. 2). In both NS groups, we observed large positive (All *d’s* > 0.8) effect sizes indicating SA expansion in the left parahippocampal gyrus and large negative (All *d’s*<−0.8) effect sizes indicating SA decreases in left caudal middle frontal gyrus, relative to the TD group (Fig. 3).

### Noonan Syndrome Is Linked To Cortical Thickness Reductions In The Precentral Gyrus And Parahippocampal Regions

We observed reductions in CT in bilateral precentral gyri and parahippocampal regions and increases in CT in lateral aspects of the occipital and frontal lobes in *PTPN11* relative to the TD group (All FDR *p*-values < 0.05) (Table S2; Fig. 2). We observed large positive effect sizes (All *d’s* > 0.7) in the left lateral occipital cortex, indicating increases in CT in both NS groups relative to the TD group. Conversely, we observed medium to large negative effect sizes (All *d’s*<−0.5) in the left parsopercularis and superior temporal gyrus as well as in the medial aspect of the temporal lobe, bilaterally, in both NS groups compared to the TD group. In affected regions, *SOS1* displays larger effect sizes relative to *PTPN11*, indicating that *SOS1* might have a more pronounced effect on CT (Fig. 3).

Due to the group differences detected in cumulative Euler number means across groups, we conducted a sensitivity analysis. After repeating the GMV, CT, and SA analyses between the *PTPN11* and TD groups with cumulative Euler number as a covariate, we observed overwhelmingly similar results relative to our initial analyses without this covariate. A subset of CT measures (in left inferior parietal, left middle temporal, left superior parietal, left supramarginal, right parahippocampal, and right superior temporal regions) and SA measures (in left precentral, left superior temporal, left insula, and right superior parietal regions) remained different (nominal *p* < 0.05) but did not survive after FDR correction.

### Convergence Effect Of Ns Subgroups On Brain Anatomy

To test whether NS subgroups have converging effects on subcortical and cortical measures, we compared maps of brain changes in *PTPN11* vs. *SOS1*. First, we calculated effect sizes and confidence intervals for each ROI and visually contrasted them (Fig. 1b; Fig. 3a). The results display the more extensive effect of *PTPN11*, relative to *SOS1*, on neuroanatomy. Next, we tested *PTPN11* and *SOS1* effect size relationships for subcortical and cortical GMV, SA, and CT^25^. Pearson correlations indicated statistically significant spatial coherence between *PTPN11* and *SOS1* on subcortical (*r* = 0.75, *p* < 0.05) and cortical GMV (*r* = 0.57, *p* < 0.001), SA (*r* = 0.63, *p* < 0.001), and CT (*r* = 0.45, *p* < 0.001). Permutation testing confirmed that observed correlations between NS subgroups (All permutation *p’s* < 0.001; Fig. 3c) are significantly greater than null expectations, indicating converging effects of NS subtypes on neuroanatomy.

#### Higher gene expressions of PTPN11 are related to larger effects of NS on surface area

To explore the relationship between genetics and neuroanatomy in the *PTPN11* group, we correlated *PTPN11* gene expression and SA effect size (Fig. 5a). *PTPN11* expression positively correlated with SA effect size at the whole-brain level (*r* = 0.32, *p* = 0.0086). Following correction for spatial autocorrelation with the null-spatial model, we detected a significant difference between the null model and observed correlation (*p*_null−spatial_=0.010), indicating that this relationship displays spatial specificity^22,27^. We also found significant differences between the null-random-gene and null-brain-gene models and the observed correlation (*p*_null−random−gene_=0.026, *p*_null−brain−gene_=0.027), suggesting that the association between SA effect size and *PTPN11* expression association is unique to the *PTPN11* gene in comparison to genes randomly selected from either the entire Allen Human Brain Atlas (AHBA) gene set or subset of these genes that are overexpressed in the brain^22^. In a subsequent lobe-wise analysis, we found that the temporal lobe (*r* = 0.54, *p* = 0.022), in particular, is driving these results (Fig. 5b–c). Furthermore, since SA effect size describes differences in SA between *PTPN11* and TD groups in specific regions, these results suggest that the higher *PTPN11* expression is in a given region, the larger the SA in children with *PTPN11* compared to the TD group. Hence, in NS, higher *PTPN11* expression is associated with larger SA. We did not find significant correlations between *PTPN11* expression and effect sizes of GMV or CT in both the *PTPN11* and TD groups.

#### Striatal volumes correlate with attentional measures in the PTPN11 group

**Figure 4:**
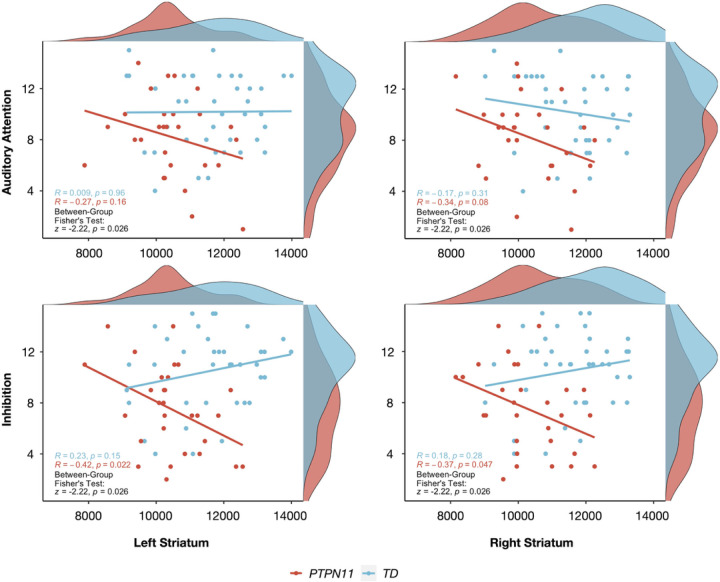
Disruption of normative anatomical-behavioral relationships relevant to attention and inhibition in Noonan syndrome. Pearson correlations between Auditory Attention or Inhibition scores and striatal volumes (in mm^3^) for each group and Fisher’s Exact Tests (with FDR corrected *p*-values) assessing differences between *PTPN11* and TD groups in brain-behavioral correlations. Participants are represented by individual dots and distribution plots of the data are displayed on the outer x- and y-axes.

**Figure 5:**
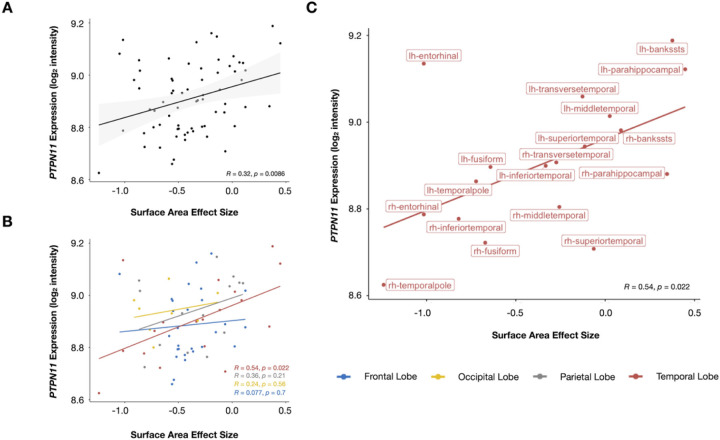
*PTPN11* expression positively correlated with surface area effect size. (A) Whole-brain, (B) lobe-wise, and(C) temporal lobe-specific correlation analyses between *PTPN11* expression and surface area effect size, which quantifies group differences in surface area between the *PTPN11* and TD groups. Individual dots represent cortical regions and dot colors denote which lobe the region is part of. Regression lines between surface area effect size and *PTPN11* expression (log_2_ intensity) for the whole brain (A: black, B: blue (frontal lobe), yellow (occipital lobe), gray (parietal lobe), red (temporal lobe), and C: red (temporal lobe)). Shaded areas in (A) represent 95% confidence intervals.

To test whether NS and TD groups differ in brain-behavioral correlations, we focused on the *PTPN11* group, given that the larger cohort provides greater power to detect differences. We tested cognitive-behavioral measures involving attention and memory as difficulties in these domains have previously been implicated in NS^24,28^. For neuroanatomical measures, we examined volumes of the striatum, which is involved in ADHD pathophysiology through the fronto-striatal pathway, and of the hippocampus, which is involved in memory^29^. Compared to the TD group, we found that *PTPN11* performed worse on Auditory Attention (*t*(54.7)=−2.59, *p* = 0.012), Inhibition (*t*(56.4)=−3.81, *p* = 0.00034), Memory for Faces Delayed (*t*(49.9)=−3.34, *p* = 0.0016), Narrative Memory Free Recall (*t*(53.2)=−3.11, *p* = 0.0030), and Narrative Memory Free and Cued Recall (*t*(55.1)=−3.23, *p* = 0.0021) domains (Table 1). Given that a sizeable proportion of the *PTPN11* (20%) and SOS1 (50%) groups were taking stimulant medications and that stimulants have been shown to improve performance on attentional measures in children with ADHD^30^, it is possible that differences between NS and TD groups on Auditory Attention and Inhibition scores may be even larger without the effect of stimulants. Bilateral striatal volumes negatively correlated with Inhibition scores in *PTPN11* with its correlation coefficient differing significantly from the TD group’s (Left: *r*=−0.42, *p* = 0.022, TD: *r* = 0.23, *p* = 0.15, Fisher test: *z*=−2.69, *p* = 0.016; Right: *r*=−0.37, *p* = 0.047, TD: *r* = 0.18, *p* = 0.28, Fisher test: *z*=−2.22, *p* = 0.026) (Fig. 4). Finally, in both the *PTPN11* and TD groups, there were no significant correlations between bilateral striatal volumes and Auditory Attention scores or bilateral hippocampal volumes and memory measures. Finally, no correlations were found after repeating these analyses without controlling for TBV. These findings confirm that brain-behavioral relationships are not driven by TBV differences between groups.

## Discussion

In this study, we sought to investigate the effect of NS pathogenic variants, which disrupt the Ras-MAPK pathway, on the developing human brain^3^. We detected decreased volumes in the bilateral corpus striatum, precentral gyri, entorhinal cortices, and superior parietal cortices—brain regions linked to attention or memory^31–33^. In the cortex, we detected corresponding NS effects on SA and CT. Specifically, we observed SA decreases in bilateral entorhinal and left superior parietal regions, and CT reductions in bilateral precentral gyri in NS relative to TD. Further, in the gene-brain analysis, we explored the link between genetics and these anatomical changes in NS, observing a positive correlation between *PTPN11* expression and SA effect size in the temporal lobe. Finally, relative to TD children, children with NS are at higher risk for attentional and memory deficits as well as comorbid diagnosis of ADHD^24^. In our cohort, 43% of the *PTPN11* group and 60% of the *SOS1* group met diagnostic criteria for ADHD (Table 1). Thus, we examined the relationship between brain anatomy and cognitive-behavioral performance in attention and memory-related tasks, which revealed decoupling of normative striatal anatomical-cognitive relationships in *PTPN11*.

In line with our previous preliminary findings^9^, we found reductions in bilateral striatum volumes in *PTPN11* relative to controls. Based on preliminary data^9^, we hypothesized that NS affects the parietal and frontal lobes. Indeed, we observed reductions in bilateral precentral gyri GMV, with corresponding decreases in CT in those regions, and bilateral superior parietal cortex GMV in *PTPN11* relative to the TD group. Decreased SA in the left superior parietal cortex and decreased CT in the right superior parietal cortex accompanied overall GMV reduction in the superior parietal cortex. The precentral gyrus and superior parietal cortex are components of the dorsal frontoparietal attention cortical network, which is involved in visuospatial attention^32,34^. Reductions in the precentral gyrus and superior parietal cortex volumes suggest that NS may disrupt the dorsal attention network. This hypothesis is supported by findings from prior studies implicating the involvement of superior aspects of the posterior parietal cortex in attentional processing^35–40^. Poorer performance in attentional measures and greater risk of ADHD diagnosis in children with NS provides further support to this potential explanation^24^. Finally, children with neurofibromatosis type-1 (NF1), another RASopathy associated with ADHD symptoms, displayed decreased precentral gyrus gray matter density relative to healthy controls^3,41^.

This study is the first to investigate brain anatomy in *SOS1*. Given the preliminary nature of the results, we used effect sizes to compare anatomical differences between NS subgroups and the TD group. Overall, we observed aberrations in GMV, SA, and CT in a similar subcortical and cortical regional distribution in both NS subgroups compared to the TD group (Fig. 3). This pattern is consistent with molecular mechanisms of *PTPN11* and *SOS1* variants, which have an activating effect on the Ras-MAPK pathway, suggesting that both NS variants similarly affect brain development and, therefore, result in aberrant anatomy^3^. For example, in both groups, we observed the greatest GMV reduction in the left (*PTPN11*: *d*=−0.90, *SOS1*: *d*=−0.80) and right pallidum (*PTPN11*: *d*=−0.88, *SOS1*: *d*=−1.03). The current study demonstrates the effect of *PTPN11* variants on the human brain and suggests that this effect extends to a second type of NS pathogenic variant in the *SOS1* gene.

In our gene-brain analysis, we observed positive correlations between *PTPN11* expression and SA effect sizes (capturing the magnitude of *PTPN11* effect on SA) on a whole-brain level and, per lobe-wise analyses, in the temporal lobe. These findings suggest that, in NS, increased SA is associated with regions with higher *PTPN11* expression and therefore, increased levels of constitutively active SHP2 mutant^3^. It is possible that the activating effect of SHP2 mutant on the Ras-MAPK pathway leads to dysregulation of neuronal and astroglial differentiation and proliferation, causing subsequent changes in anatomical measures, such as SA, in the *PTPN11* group^42^.

Our results are limited by availability of transcriptomic data in brain tissue for individuals with NS. We utilized *PTPN11* expression levels from typically developing adults as a proxy for data from individuals with NS^16^. Although it is possible that *PTPN11* expression levels from adult brains may not have equivalent regional mapping in those of children within our cohort’s age range, we decided to utilize AHBA as it is the most comprehensive dataset of the cortical transcriptome to date. Further details regarding our utilization of AHBA data are provided in Supplementary Material.

To test our hypothesis that the effects of the Ras-MAPK pathway on striatal structure are central to attentional functioning, we performed brain-behavior correlations. First, we found differences between *PTPN11* and TD groups in correlations of bilateral striatal volumes and measures of inhibition, a key aspect of executive function and attention. Second, in the *PTPN11* group only, we found a negative correlation, indicating that smaller bilateral striatal volumes are associated with better performance on inhibition tasks. Third, we found that correlations differ between *PTPN11* and TD groups. Given that we observed reduced striatal volumes in children with NS, we expected inhibition task performance to positively correlate with increasing striatal volume. It is possible that altered and more efficient brain reorganization, resulting from recruitment of other brain structures, leads to smaller striatal volumes and better performance in some children with *PTPN11*. Our hypothesis-based approach to querying brain-behavior relationships is supported by evidence of the large effects of NS on striatal volumes and inhibition^9,24^. However, this approach has significant limitations, as evidenced by studies conducted in larger (yet non-clinical) populations such as the *Lifespan Human Connectome Project in Development study*^43^ or *Adolescent Brain Cognitive Development* (*ABCD)* Study^44,45^. Future data-driven studies utilizing large cohorts of rare genetic conditions such as in the case of 22q11.2 deletion syndrome in the Philadelphia Neurodevelopmental Cohort (PNC) datasets^46^ are warranted for the Rasopathies.

Memory deficits have previously been implicated in mouse and human models of NS^24,47^, particularly in verbal long-term memory, which is dependent on the hippocampus. Here, we describe, for the first time in humans, a reduction in right hippocampal GMV in *PTPN11* relative to the TD group. In the temporal lobe, reductions in bilateral entorhinal cortex SA contributed to decreased bilateral entorhinal cortex GMV in the *PTPN11* group. Taken together, reduced volume in the right hippocampus and bilateral entorhinal cortices, which provide substantial afferent projections to the hippocampus, in *PTPN11* provide further evidence that NS disrupts hippocampal circuitry^31^.

Our striatal and cortical findings suggest that NS has an effect on structures in frontostriatal circuits, which have been implicated in ADHD^48^ as well as in developmental language disorders and motor impairments^49,50^, which are frequently found in NS and other RASopathies^49–51^. The presence of these conditions, in addition to attentional problems, in children with NS, supports the notion that these pathways are involved in several cognitive processes.

This study suggests a new framework of investigating NS and its effect on the brain that is translational in two senses: in examining findings from mouse models of NS in humans and in exploring the interactions between brain anatomy, behavior, and genetics. It is a first step in investigating the impact of NS genotypes (*PTPN11* and *SOS1*) on brain anatomy. Studying the effect of these single gene disorders disrupting specific steps in Ras-MAPK pathway on brain development can elucidate the pathophysiology of the RASopathies. The *SOS1* subgroup has a sample size limitation (*n* = 10); thus, the results from the *SOS1* analysis should be considered in the context of small sample size. Although it is preliminary, the *SOS1* analysis provides new information on the anatomical-behavioral phenotype of the *SOS1* variant and offers an unprecedented opportunity to compare two pathogenic variants associated with NS (*PTPN11* and *SOS1*). Future studies with larger sample sizes for *SOS1* and longitudinal investigation of neurodevelopmental trajectories in children with both NS genotypes could expand upon our findings. Our exploratory gene-brain and brain-behavior analyses offer new information regarding genetic influences on anatomy, and, in turn, anatomical influences on cognition and behavior in NS and the RASopathies. Finally, our results increase our understanding of the effects of the Ras-MAPK pathway on the human brain and the neural underpinnings of neurodevelopmental disorders associated with the RASopathies. Given that MEK inhibitors, Ras-MAPK-pathway-altering medications, are already in use for children with RASopathies^52,53^, these insights are more essential than ever.

## Figures and Tables

**Table 1:** Participant Demographic and Medical Information

	*PTPN11*	*SOS1*	Typically Developing	*p* value^a^	*p* value^b^
Number of participants	30	10	40	-	-
Sex (n)	Female (19)	Female (6)	Female (27)	ns	ns
	Male (11)	Male (4)	Male (13)	ns	ns
Age range	4.43–12.3	5.71–10.34	4.05–11.94	-	-
Tanner Stage	≤ 2	≤ 2	≤ 2	-	-
GH	13	0	0	-	-
Stimulants	6	5	1	-	-
SSRI	3	3	0	-	-
Mean Age	8.7 ± 2.31	7.93 ± 1.53	9.24 ± 1.62	ns	*p* < 0.05
FSIQ (WISC/WPPSI)	90.4 ± 14.53	97.2 ± 13.88	109.5 ± 7.65	*p* < 0.001	*p* < 0.05
VCI	96.17 ± 12.83	100.3 ± 11.09	112.03 ± 11.57	*p* < 0.001	*p* < 0.05
PRI	93.67 ± 12.50	101.00 ± 12.56	111.45 ± 10.77	*p* < 0.001	*p* < 0.05
WMI^c^	86.29 ± 13.39	94.33 ± 13.57	101.4 ± 8.7	*p* < 0.001	ns
PSI	85.87 ± 15.02	89.8 ± 16.78	98.26 ± 12.6	*p* < 0.001	ns
Attention Problems^d^	59.79 ± 10.38	62.50 ± 11.93	51.68 ± 10.28	*p* < 0.01	*p* < 0.05
Hyperactivity^d^	63.28 ± 14.07	63.20 ± 16.43	48.76 ± 11.93	*p* < 0.001	*p* < 0.05
Auditory Attention^e^	8.21 ± 3.19	9.8 ± 3.52	10.18 ± 2.89	*p* < 0.05	ns
Inhibition^f^	7.51 ± 3.36	7.70 ± 3.83	10.51 ± 2.99	*p* < 0.001	ns
Memory for Faces Delayed^f^	8.72 ± 3.05	10.3 ± 3.83	10.97 ± 2.29	*p* < 0.01	ns
Narrative Memory Free and Cued Recall^g^	9.17 ± 2.95	8.80 ±3.63	11.79 ±3.74	*p* < 0.01	*p* < 0.05
Narrative Memory Free Recall^h^	9.38 ±3.02	9.30 ±3.55	11.89 ±2.89	*p* < 0.01	*p* < 0.01
ADHD Diagnosis^i^	43.3% (14/30)	60.0% (6/10)	-	-	-

All values are reported in mean ± standard deviation; Welch’s two-sample *t*-test was used to assess significance between groups; GH, growth hormones; SSRI, Selective serotonin reuptake inhibitor; FSIQ, Full-scale intelligence quotient; PIQ, Performance intelligence quotient; VIQ, Verbal intelligence quotient; PSI, Processing speed intelligence; WMI, Working memory intelligence; Inhibition, Inhibition vs. Naming Contrast; ns, not significant.

a Children with *PTPN11* compared with controls

b Children with *SOS1* compared with controls

c *n* of 24 for *PTPN11*, *n* of 9 for *SOS1*, and *n* of 39 for TD for WMI given age-restrictions of respective assessments (WISC (WMI) > 6 years).

d *n* of 29 for *PTPN11*, *n* of 10 for *SOS1*, and *n* of 38 for TD

e *n* of 28 for *PTPN11*, *n* of 10 for *SOS1*, and *n* of 39 for TD, Auditory Attention not administered to children age < 5

f *n* of 29 for *PTPN11*, *n* of 10 for *SOS1*, and *n* of 39 for TD, as Inhibition (Naming vs. Inhibition Contrast) and Memory for Faces Delayed are not administered to children age < 5

g *n* of 30 for *PTPN11*, *n* of 10 for *SOS1*, and *n* of 39 for TD

h *n* of 29 for *PTPN11*, *n* of 10 for *SOS1*, and *n* of 38 for TD

i Percentage and ratio of children in each NS group meeting diagnostic criteria for ADHD in the K-SADS-PL
